# Robotic surgical curriculum for medical students: a scoping review

**DOI:** 10.1007/s11701-025-02667-w

**Published:** 2025-08-19

**Authors:** Jade El-Mohamed, MohammadAli Ahmadipour, Satish Warrier, Tony Costello, Michael Hii, Helen Mohan

**Affiliations:** 1 International Medical Robotics Academy (IMRA), Melbourne, VIC Australia; 2 St Vincent’s Health, Melbourne, VIC Australia; 3 University of Melbourne, Victoria, Australia; 4 Royal Melbourne Hospital, Melbourne, VIC Australia; 5 Peter MacCallum Cancer Centre, Melbourne, VIC Australia; 6 Alfred Health, Melbourne, VIC Australia; 7 Austin Health, Heidelberg, VIC Australia

**Keywords:** Robotic surgery, Training and education, Medical students, Curriculum design

## Abstract

**Background:**

Robotic surgery is an increasingly common component of surgical practice, yet it remains underrepresented in medical student education. While student interest in robotic surgery is high, their exposure is often informal and incidental.

**Objective:**

To map the existing literature on robotic surgery curricula developed for medical students and to evaluate the extent to which these curricula align with Kern’s six-step framework for curriculum development.

**Methods:**

A scoping review was conducted using PRISMA-ScR guidelines. MEDLINE, EMBASE, and Cochrane databases were searched. Studies were included that focused on medical students and addressed one or more of Kern’s six curriculum development steps. Dual independent screening and consensus coding were used. Data were extracted and mapped to Kern’s framework.

**Results:**

Twenty-one studies were included, primarily small, single-institution pilots featuring the da Vinci surgical system. Most described technical skills training delivered via simulation. Few studies defined learning objectives and none addressed non-technical skills such as communication and teamwork. Implementation was limited with no evidence of long-term evaluation or curricula integration.

**Conclusion:**

While robotic surgery curricula for medical students are feasible and valued, they remain in early stages of development. There is a need for structured, scalable and educationally grounded curricula that introduce foundational knowledge and support student readiness for technology-integrated surgical practice.

## Introduction

Robotic surgery is transforming operative care across numerous specialties. As this technology becomes increasingly integrated into surgical practice, it raises a fundamental question: should all medical students be exposed to robotic surgery, and if so, how? Some argue that all future doctors should develop foundational understanding, enough to safely participate in surgical teams and counsel patients [1]. Others argue that students should spend more time with people and less with robots [2].

This debate comes at a time of rapid change. Robotic platforms are proliferating, costs are falling and indications are expanding [3–8]. Robotic-assisted procedures are now routine across many surgical units, including in Australia, where graduate and undergraduate-entry programs train large and diverse cohorts. Yet most medical students remain unexposed to robotic systems or encounter them only passively [9, 10]. Their knowledge is often self-directed or acquired by chance, and learning in the robotic theatre is described as demotivating [11].

What is striking is not just a lack of access, but a lack of intentional design [12]. While robotic training is increasingly available to surgical trainees and fellows [13], few programs have positioned medical students as the intended learners. Instead, students are often recruited as participants in simulator studies or curriculum validation [14–19]. This review takes the position that this represents a missed opportunity to engage, orient and prepare future doctors for the surgical environment where robotic systems are standard.

Importunately, this is not about teaching students to operate. It is about designing developmentally appropriate curricula that introduce core concepts, promote technological fluency and support broader professional growth. For Australian students, some of whom enter medicine directly from high school, early exposure may serve as a platform for exploration, motivation and informed career choice. Structured curricula can also support students in gaining non-technical skills essential to robotic care, such a situational awareness, communication and teamwork.

Despite strong student interest [20–23], efforts to implement robotic curricula at the medical student level remain fragmented. Most initiatives are institution-specific and focus heavily on technical simulation. There is limited guidance on curriculum content, delivery strategies, or alignment with student learning needs. A shared framework is required to guide this work.

**Fig. 1:**
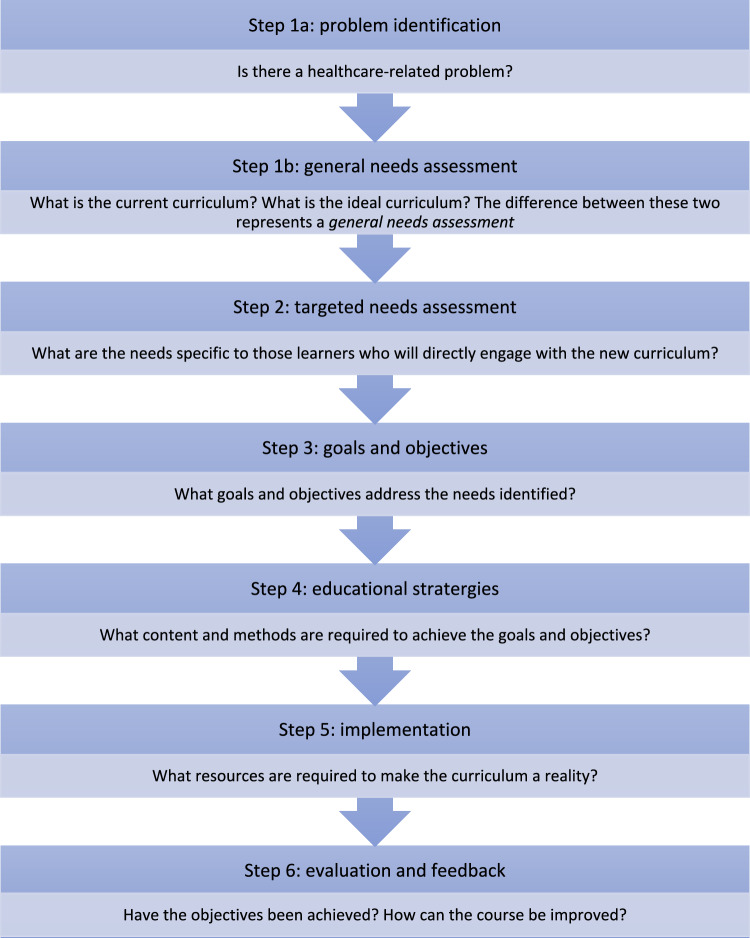
Kern’s Six Step Approach to Curriculum Development [24]

This scoping review aimed to map existing literature on robotic surgical curricula developed for medical students, with a focus on how these initiatives align with Kern’s six-step framework for curriculum development [24]. Kern’s model (outlined in Fig. 1) provides a structured, staged approach to curriculum design and served as an analytical framework to assess which components of the development process were addressed in existing literature.

Through this lens, we sought to clarify what currently exists, highlight gaps in curriculum structure and intent, and inform the future design of effective, level appropriate robotic surgical education at the medical student level.

## Methods

Given the paucity of data on this topic, scoping review methodology was adopted. This review used the Preferred Reporting Items for Systematic reviews and Meta-Analyses extension for Scoping Reviews (PRISMA-ScR) guidelines. The review protocol was developed in advance but was not registered.

### Eligibility criteria

Studies were included if they:

- Were peer-reviewed and published in English
- Focused on robotic surgery in the context of medical student education
- Included participants defined as undergraduate or graduate-entry medical students
- Addressed at least one of Kern’s [24] six steps of curriculum development

Studies employing quantitative, qualitative and mixed-methods approaches were included to capture diverse facets of medical student education.

Studies were excluded if they:

- Used medical students to validate curricula for vendors, trainees or surgeons
- Included mixed cohorts without disaggregated data for medical students

### Information sources and search strategy

A comprehensive literature search using the following bibliographic databases: MEDLINE, EMBASE and the Cochrane Database of Systematic Reviews and Central Register of Controlled Trials. Manual citation chasing was also performed using the reference lists of included studies. The search was conducted in April 2024, with no restrictions applied to the earliest publication date.

The search strategy was developed in consultation with an academic librarian and combined both MeSH terms and free-text keywords to maximise sensitivity. Search terms related to robotic surgery and medical student education were structured as follows:

- Population terms: "medical student*", "Students, Medical", "Education, Medical, Undergraduate", "undergraduate*"
- Intervention terms: "Robotic Surgical Procedures/ed", "Robotics/ed", "robot* surg*", "robot*", "surg*"
- Combined searches: Medical student terms were intersected with robotic surgery and surgical education terms using Boolean operators (e.g., AND/OR).

The full MEDLINE search strategy is provided in Appendix 1.

### Selection process

Two reviewers (J.E. and M.A.) independently screened the titles and abstracts of all retrieved records, followed by full-text assessments. Discrepancies were resolved through discussion and consensus.

### Data extraction and analysis

A data charting form was jointly created to define the variables of interest. Each reviewer extracted data separately, after which they met to reconcile differences and finalise the results. Extracted data included general study characteristics, participant details, outcomes and educational themes. A template of this charting form can be found in Appendix 2.

Each study was then mapped to Kern’s [24] six steps for curriculum development: problem identification, targeted needs assessment, goals and specific objectives, educational strategies, implementation, evaluation and feedback. Key information relevant to informing a robotic surgical curriculum for medical students was highlighted under each domain.

## Results

**Fig. 2:**
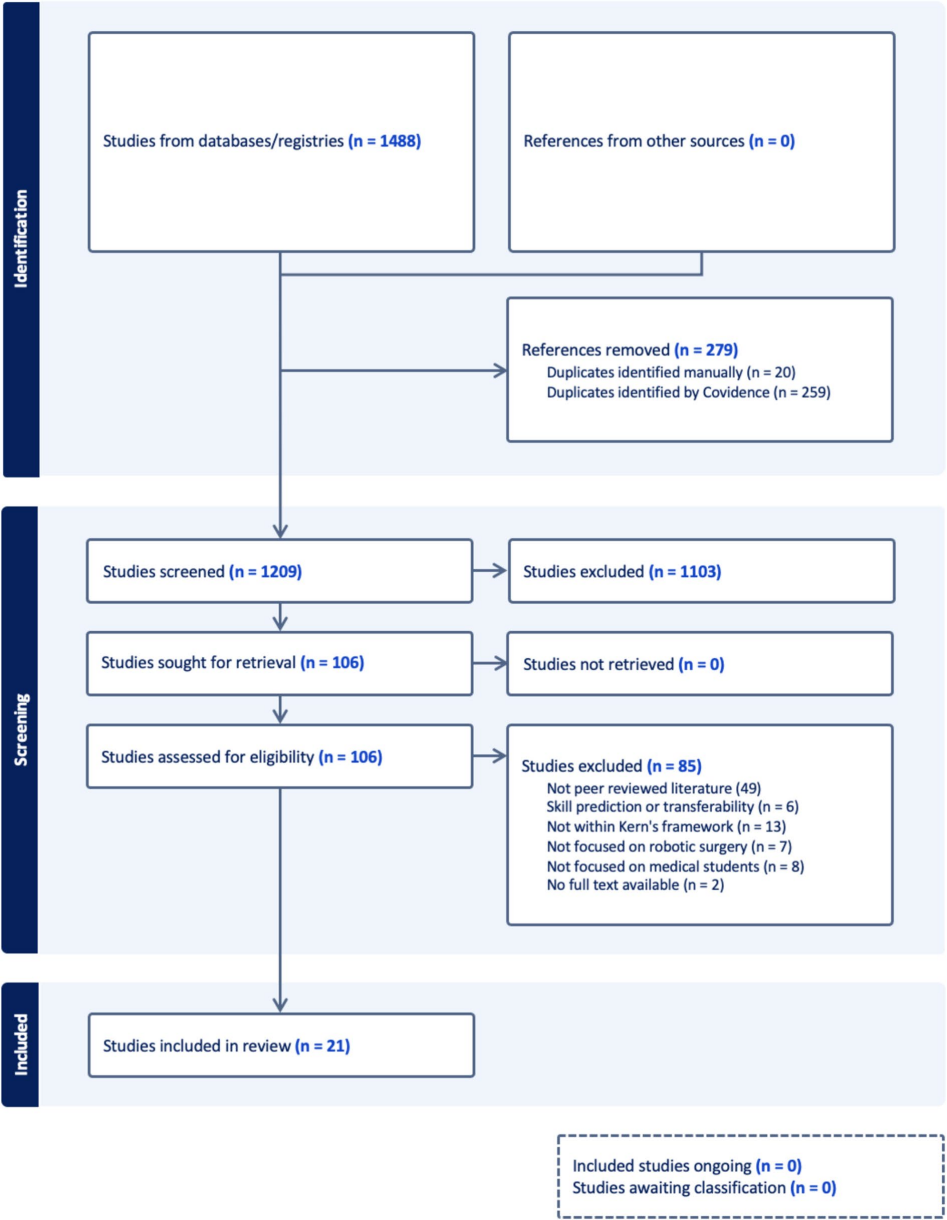
PRISMA-ScR Flowchart

The search strategy retrieved 1488 publications. After removing duplicates, 1209 titles and abstracts were screened. Of these, 106 full-text articles were reviewed and 21 studies met the inclusion criteria. No additional articles were identified through citating chasing (Fig. 2).

Study designs included randomised controlled trials, cohort studies, cross-sectional surveys, qualitative research and one narrative description. There were no systematic reviews or meta analysis. One study “Perception of robotic-assisted surgery among medical students: a systematic review and meta-analysis” [25], was excluded as its analysis focused exclusively on residents and fellows. Most studies were small, single-institution initiatives focusing on the da Vinci surgical system or related simulators.

Table 1 maps each study to Kern’s six-step framework for curriculum development. Table 2 outlines key study characteristics. Table 3 summarised findings relevant to informing medical student-focused curricula.

**Table 1:** Mapping of Included Studies to Kern’s Six-Step Curriculum Development Framework

Author	Publication Year	Kern’s Framework
Newland et al. [10]	2024	Problem Identification and General Needs Assessment; Targeted Needs Assessment; Educational Strategies; Implementation; Evaluation and Feedback
Ekrutt et al. [22]	2022	Targeted Needs Assessment
Garcia-Chavez et al. [26]	2023	Targeted Needs Assessment
Higgins et al. [11]	2020	Targeted Needs Assessment
Sighinolfi et al. [21]	2023	Targeted Needs Assessment
Sultan et al. [9]	2022	Targeted Needs Assessment; Goals and Objectives
Summers et al. [29]	2014	Goals and Objectives; Educational Strategies
Mullens et al. [27]	2021	Goals and Objectives; Educational Strategies
Greenberg et al. [28]	2023	Goals and Objectives; Implementation; Evaluation and Feedback
Benson et al. [30]	2010	Educational Strategies
Chiu et al. [31]	2019	Educational Strategies
Gurung et al. [42]	2019	Educational Strategies
Harris et al. [35]	2017	Educational Strategies
Kang et al. [33]	2015	Educational Strategies
Laca et al. [37]	2022	Educational Strategies
Melnyk et al. [36]	2021	Educational Strategies
Shim et al. [34]	2018	Educational Strategies
Suh et al. [32]	2011	Educational Strategies
Van der Leun et al. [38]	2022	Educational Strategies
Kalinov et al. [40]	2023	Educational Strategies; Evaluation and Feedback
Moglia et al. [39]	2018	Implementation

The classification of each study to Kern’s framework was conducted independently by two reviewers and finalised through consensus. While several studies coded as “Targeted Needs Assessment” also identified broader system-level gaps (e.g. lack of exposure, limited institutional access), we retained the original classification to preserve methodological integrity. These overlapping themes are discussed in the Results and Discussion narratives

**Table 2:** Study Characteristics

Author	Country	Aims	Study Design	Population	Participant Number	Outcomes
Benson et al. [30]	United States	Assess the impact of structured (formal) versus unstructured (informal) instruction on the development of robotic surgical skills among novice surgeons	Randomised controlled trial	Medical students with no prior exposure to robotic surgery	43	Formal instruction enhanced novice surgeons' performance in complex robotic tasks, but showed no significant effect on simpler task execution
Chiu et al. [31]	Taiwan	Investigate whether incorporating peer observation alongside expert demonstration enhances performance in virtual reality-based tasks	Cohort study	Medical students from Taipei Medical University naïve to robotic surgery	50	Performance in intermediate simulator tasks is positively correlated with active engagement
Ekrutt et al. [22]	Germany	Evaluate medical students’ initial interest in robotic-assisted surgery and explore whether participation in a hands-on robotic training course influences their interest in pursuing a surgical career	Randomised controlled trial	Medical students	100	There is a strong interest in integrating robotic training into medical student education Hands on robotic training increases attractiveness and interest in the field of surgery
Garcia-Chavez et al. [26]	United States	Investigate the influence of early exposure to robotic surgery on: 1) medical students’ interest in a surgical career, and 2) their performance in simulation-based tasks	Cohort study	Medical students, mostly in their pre-clinical years, at the University of Florida College of Medicine	17	Introducing medical students to robotic surgery: 1) enhances their interest in pursuing a surgical career 2) leads to measurable improvements in technical skills
Greenberg et al. [28]	United States	Evaluated the practicality of implementing simulation-based training through a pilot course designed for medical students	Qualitative research	Medical students across all years at a single institution All had been exposed to robotic surgery	14	The pilot program was successfully implemented and well-received by the target participants. Its value as a preliminary step toward broader curricular integration was acknowledged Hands- on simulation increases the preparedness of medical students to enter and learn in a robotic operating room
Gurung et al. [42]	United States	Determine if an accelerated skills acquisition protocol (ASAP) 1) is more efficient in robotic simulator skill acquisition 2) maintains skill transfer and retention as compared to a conventional proficiency based training protocol	Randomised controlled trial	Undergraduate medical students without prior robotic surgical training	16	The ASAP group reached proficiency with fewer practice attempts and training sessions Once proficiency was acquired it was equally transferable and similarly retained
Harris et al. [35]	United Kingdom	Determine if observing expert, novice or mixed models improved skill acquisition on a surgical simulator	Randomised controlled trial	120 undergraduate medical with no previous robotic surgery experience	120	Comparable benefits were observed whether students watched a traditional expert demonstration or a model that included deliberate errors
Higgins et al. [11]	United States	Determine 1) medical student perceptions of robotic surgery during clinical placement 2) if the operating room environment provides motivation for learning	Qualitative research	Senior medical students at the Medical College of Wisconsin who encountered robotic surgery during their clinical clerkships	10	Perceptions: robotic surgery creates a prominent physical presence in the clinical setting, offering enriched learning opportunities through its immersive visual and auditory environment Motivation: the robotic theatre is a demotivating and a monotonous learning environment for students
Kalinov et al. [40]	Bulgaria	Examine the level of satisfaction among medical students following robotic surgery simulator training	Qualitative research	Medical students at the University of Varna, Bulgaria	30	The robotic simulator is an easy to use and useful training tool Exposure to the simulator increased students’ interest in pursuing further training in robotic surgery
Kang et al. [33]	South Korea	Determine the most effective simulator training schedule	Prospective non-randomised study	Medical students without experience using surgical robots	30	Shorter intervals of training are more effective in improving skill acquisition
Laca et al. [37]	United States	Examine the impact of standardised, real-time feedback on performance during a simulated dissection task	Randomised controlled trial	Medical students with no prior surgical experience	45	Feedback decreased students error rate and increased their technical performance Students who initially demonstrated lower performance showed the greatest improvement following feedback
Melnyk et al. [36]	United States	Evaluate whether robotic simulation training incorporating expert gaze guidance enhances student performance more effectively than conventional movement-based training methods	Randomised controlled trial	Medical students without prior robotic experience	18	Gaze-augmented training improves movement efficiency by promoting the adoption of expert gaze strategies, which remain effective even under added stressors The benefits of gaze-augmented feedback are most evident during the early stages of training
Moglia et al. [39]	Italy	Evaluate the practicality of introducing simulation-based training into medical student education Describe how medical students progress in skill acquisition when using virtual reality surgical simulators	Cohort study	Undergraduate medical students from the University of Pisa	5	Simulation-based program is feasible Student with prior experience in simulation required less attempts to reach proficiency
Mullens et al. [27]	United States	Describe a robotic surgery elective curriculum for senior medical students	Narrative	Senior medical students	N/A	N/A
Newland et al. [10]	United States	Assess the perspectives and experiences of early-stage surgical trainees regarding robotic surgery	Qualitative research	First- and second year surgical residents and fourth- year medical students	85 trainees (58 residents and 27 medical students)	A notable educational gap remains, as early exposure to robotic surgery continues to be limited despite growing demand
Shim et al. [34]	South Korea	Determine if expert proctoring, educational video or independent training is most effective in the acquisition of robotic surgical skills using a simulator	Randomised controlled trial	Robotic naïve medical students	45	Self-directed training appears less effective compared to other instructional approaches Educational videos may offer comparable benefits to direct expert supervision
Sighinolfi et al. [21]	Italy	1) Evaluate the level of knowledge and interest in robotic surgery among nursing and medical students 2) Examine the effect of a hands-on training course utilising the Hugo RAS and Versius systems on learner outcomes	Cross-sectional study	Undergraduate medical and nursing students	41 (23 nursing and 18 medical)	Hands on exposure increases attractiveness A large majority (95%) of medical students expressed a desire for a formal robotic surgery course to be included in their curriculum
Suh et al. [32]	United States	Develop a systematic and validated training program for robotic laparoscopic skills that includes objective evaluation tools to assess learner performance	Cohort study	Medical students from the University of Nebraska Medical Center with no prior experience using a surgical robotic system	15	The structured 4-day program increased objective and subjective performance on the surgical robot Learning was retained after 1-day
Sultan et al. [9]	Saudi Arabia	Investigate medical students’ understanding of and attitudes toward robotic surgery	Cross sectional study	Medical students from multiple institutions across Saudi Arabia	239	Medical students demonstrate a knowledge gap regarding robotic surgery, relying primarily on the internet as their main source of information. Nevertheless, they exhibit a positive attitude and hold high expectations for the technology
Summers et al. [29]	United States	1) Evaluate the effectiveness of a standardised curriculum in teaching basic robotic skills to novice learners using the da Vinci platform 2) Compare the outcomes of structured practice sessions versus self-directed, unstructured practice for acquiring dry lab skills	Randomised controlled trial	Medical students from the Loyola University Chicago Stritch School of Medicine	17	1) Novice surgeons can enhance their robotic surgical skills through a structured program that includes orientation, guided introduction, proctoring, and dry-lab exercises 2) Self-directed practice appears to yield comparable outcomes to those achieved through a structured, objective-driven curriculum
Van der Leun et al. [38]	Netherlands	1) Examine the effect of video-based feedback on the acquisition of robotic surgical skills by novice surgeons training with a virtual reality simulator 2) Determine the extent to which skills learned on a robotic simulator translate to competent performance on a real robotic surgical platform	Randomised controlled trial	Medical students and medical-PhD candidates from the University Hospital of Utrecht	40	Video review enhances the quality of robotic surgical skills in novices Simulator skills are transferable from the simulator to robotic platforms

**Table 3:** Key Findings to Inform a Curriculum

Author	Key information to inform curriculum
Needs Assessment	
Ekrutt et al. [22]	There is a demand for implementation of an up-to-date robotic surgery curriculum in medical education Hands-on training is important in increasing positive attitudes towards RAS in medical students
Garcia-Chavez et al. [26]	Medical students show considerable enthusiasm for early exposure to robotic surgery and are keen to engage in hands-on experiences
Higgins et al. [11]	Improving the educational value of the robotic operating room requires a curriculum that includes structured orientation, meaningful student roles, and simulation-based learning
Sighinolfi et al. [21]	Exposure to hands-on robotic surgery simulation increases student interest in robotic surgery and should be included in a curriculum where feasible
Sultan et al. [9]	Although their knowledge of robotic surgery is limited, students are interested in the field. The curriculum should equip them with essential knowledge for future clinical decision-making, including making appropriate referrals. Educational content should address clinical, technical and ethical aspects of robotic surgery to ensure a well-rounded understanding
Goals and objectives	
Greenberg et al. [28]	Course objectives included introducing students to robotic features, functionalities and roles Students value hands-on, clinically relevant training. They prefer active, rather than didactic learning, desire time on the robotic console and real-time guidance
Mullens et al. [27]	Goals of this curriculum: - Orient students to robotic surgical technology - Understand the application of robotics in surgical subspecialties - Prepare students for resident-level training Components of curriculum included: - Online training - Simulator curriculum - Hands-on robotic training - Practical exposure in the operating room
Educational strategies	
Benson et al. [30]	Learning the robotic skills to perform simple tasks does not seem to require expert surgeon guidance. However, this guidance is necessary in learning the technical skills required for the successful completion of complex tasks
Chiu et al. [31]	Peer observation increases the efficiency with which intermediate simulators tasks can be learned
Gurung et al. [42]	Learning robotic simulator skills need not occur in a stepwise fashion. An accelerated curriculum results in expedited skill acquisition without compromising skill transferability or retention
Harris et al. [35]	It is equally beneficial for the acquisition of technical skill to view either error-sewn novices or experts perform a surgical task. This information may help in reducing the resourcing requirements for delivering a robotic surgical curricula
Kang et al. [33]	Regular short-interval training (daily for 1h) is more effective in skill acquisition than longer interval (1h each week) training. Intensive long-duration practice (4h in one day) is the least effective training schedule
Laca et al. [37]	Contemporaneous feedback improves technical performance. This is particularly helpful for students who initially struggle
Melnyk et al. [36]	Understanding expert gaze patterns (focusing on target rather than instruments) can increase the efficiency of technical skills acquisition
Shim et al. [34]	Educational video is as useful as expert proctoring in terms of technical skills acquisition on a robotic surgical simulator. It would be beneficial to develop a standardised educational video
Suh et al. [32]	A structured training program is an effective method to teach novices fundamental robotic surgical skills
Summers et al. [29]	Development of a structured curriculum results in improved technical skill. Self-directed practice as effective as structured, objective-oriented curriculum
Van der Leun et al. [38]	Video review of own performance followed by an expert performance enhances robotic surgical skills
Implementation	
Moglia et al. [39]	Simulation-based program is feasible. Expect students with prior simulation experience to perform better
Newland et al. [10]	Implementing a structured, multidisciplinary robotic surgery curriculum early in training is feasible. It has the potential to address current educational gaps, promote greater familiarity and confidence with robotic systems, and ultimately enhance efficiency in the clinical environment
Evaluation and feedback	
Kalinov et al. [40]	Robotic surgery simulator training is an acceptable and appreciated educational method for medical students

### Medical student interest and identified gap in robotic education

#### (Mapped to: Problem Identification and Needs Assessment)

Several studies identified a disconnect between the increasing prevalence of robotic surgery in clinical practice and its limited representation in medical school curricula [1–5]. This disparity was described as a pressing issue by both students and educators [9, 10]. Students reported having little formal exposure and acquiring knowledge opportunistically [9]. Higgins et al. [11] noted that the robotic operating room was often perceived as an unwelcoming and passive learning environment.

At the same time, students demonstrated strong enthusiasm for learning about robotic surgery and a clear desire for structured educational opportunities [21, 22, 26]. This tension between motivation and lack of access emerged as a key theme across studies, supporting the need for formalised, level-appropriate curriculum.

### Goals and objectives of reported curricula

Only a few studies explicitly articulated learning goals. These included orienting students to robotic technology [27], developing an understanding of clinical applications [27, 28] and equipping students with sufficient knowledge to make appropriate referrals in future practice [9]. In Mullens et al. [27], the curriculum was also intended to prepare students for subsequent resident-level training.

### Educational strategies for medical students

Educational strategies described in the literature were predominantly focused on technical skill acquisition via simulation. The most common format included an online introduction followed by in-person sessions using simulators or robotic platforms [10, 16, 27, 29–32]. To enhance skill acquisition, several studies trialled innovative strategies:

- Short interval or disturbed training schedules [33]
- Observation of expert vs. error-prone models [34, 35]
- Expert gaze-guided feedback [36]
- Video review, peer observation and real time feedback [30, 31, 37, 38]

These interventions were shown to expediate learning and improve technical performance among novice users.

### Curriculum implementation, evaluation and feedback

Studies by Greenberg et al. [28] and Moglia et al. [39] demonstrate that robotic curricula can be feasibly implemented for medical students, even on a small scale. Students expressed high satisfaction with hands-on, clinically relevant teaching and showed improved preparedness to participate in robotic operating rooms [28].

Despite these positive findings, most curricula were pilot programs limited to a single site. Kalinov et al. [40] reported positive student feedback and skill improvement, but no studies demonstrated large-scale implementation or long-term integration into formal curricula.

### Summary of findings aligned with Kern’s framework

Collectively, these studies suggest that robotic surgery is a relevant and engaging area of interest for medical students. However, current offerings remain fragmented, short-term and heavily skewed towards technical simulation. Curriculum content and goals are inconsistently defined and non-technical skills such as communication and team-based competence, remain largely unaddressed.

Multimodal, experiential teaching formats appear most effective, particularly when combined with feedback, peer interaction and performance review. The feasibility of small-scale implementation has been demonstrated, yet broader integration will require institutional support, faculty involvement and alignment with curricular priorities.

## Discussion

Robotic surgery is transforming operative care across multiple specialties at an unprecedented pace. However, this technological revolution is not adequately reflected in medical student education. While prior research has examined robotic training for surgical residents and fellows [13] our review highlights a noticeable gap. Medical students remain underrepresented as the primary learners in robotic surgical curricula. This is despite their clear recognition of robotic surgery as a central element of future practice and their expressed eagerness to engage with the technology early in training. This disconnect, where students are engaged and eager yet not positioned as the intended learners, represents a missed opportunity to cultivate early surgical interest, foster foundational understanding and prepare future doctors for technology-integrated clinical practice. Medical students feature prominently in the literature on robotic surgical education, but their involvement is often instrumental rather than intentional. Numerous studies enlist students to characterise simulator learning curves [16], assess virtual reality training platforms [14, 15], or validate curricula designed for residents and fellows [17–19]. While this provides valuable insight into educational technologies, it unconsciously positions students as proxies in research rather than as learners in their own right. This was not unreasonable as early efforts in robotic education necessarily focused on upskilling practicing surgeons and subspecialty trainees as the technology was first adopted. However, as robotic surgery becomes increasingly embedded in routine surgical care, the educational focus must evolve. It is now both timely and essential to reimagine medical students as primary learners, and to develop dedicated curricula that align with their learning needs, clinical responsibilities, and professional aspirations. This imperative is particularly relevant in the Australian context, where medical programs include both undergraduate-entry and graduate-entry pathways. As a result, students may begin training with varying levels of clinical exposure and career clarity. Structured, student-focused curricula in robotic surgery can offer a valuable platform for early exploration, foundational learning, and technological literacy. These goals differ from those prioritised in graduate-entry models, such as those in the United States.

The majority of existing robotic surgery curricula are designed with technical skill acquisition in mind. These often emphasise console time, suturing and knot-tying, which reflects the performance needs of surgical trainees and fellows [41]. However, this emphasis is less appropriate for medical students. Our review found little guidance on what learning objectives are appropriate for this cohort. Most interventions describe general exposure, orientation to the robotic platform, or basic psychomotor tasks [27–29]. While technical activities can be engaging, they may overlook broader learning goals. A student-focused curriculum should aim to build foundational understanding, spark interest, and increase comfort with robotic systems. This includes preparing students to explain robotic surgery to patients, understand the challenges, risks and benefits and feel confident in a robotic operating room. Training should align with their level of responsibility and help situate robotic surgery within the wider context of surgical care.

Many studies included in this review demonstrate that medical students can meaningfully engage with robotic surgery through structured simulation. Educational strategies such as abbreviated simulation protocols [42], active observation [31, 35], video review [38], contemporaneous feedback [37] and expert facilitation [30] have all proven effective in enhancing technical skill acquisition among students. These approaches offer scalable and efficient options that align with modern surgical education.

Technical proficiency alone is insufficient for safe robotic practice. Robotic surgery introduces unique challenges. Notably, the physical separation of the surgeon from the patient and the operative team. This disrupts traditional communication, reduces non-verbal cues, and alters team dynamics. Human factors training has therefore become an increasingly important component of robotic surgery curricula for residents and fellows. A systematic review by Mahendran et al. [43] highlighted that robotic-assisted procedures demand heightened situational awareness, clear communication, and effective teamwork. These skills are often underdeveloped yet critically linked to patient safety. The Royal College of Surgeons of Edinburgh has similarly emphasised the need for graduated training programs that integrate non-technical skills from the outset of robotic training [44]. Despite these advances, our review found no evidence that such competencies are addressed in reference to medical students. Medical students must not only gain foundational technical understanding but also begin to develop the professional behaviours and human factors that support safe surgical practice. Importantly, integrating human factors into a robotic surgery curriculum also offers a valuable opportunity to introduce these universally relevant skills to all future clinicians, not only those pursuing surgical careers. Simulation-based education, particularly when delivered in immersive scenarios with structured debriefing, remains the most effective method to teach these non-technical skills [45] and should be a priority in any medical student-focused robotic curriculum.

Several studies demonstrate that robotic surgery curricula for medical students are feasible and well-received [10, 28]. However, these efforts remain largely limited to single-institution pilots without widespread curricular integration or standardisation. Within Kern’s framework for curriculum development, these initiatives address early steps such as identifying learner needs and trialling educational strategies, but there is no literature demonstrating a progression to sustained implementation or evaluation. This reflects a broader challenge in surgical education. That is translating innovation into sustainable, curriculum-wide change. The enthusiasm expressed by medical students [21, 22, 46] and their preference for hands-on, active learning [28] create a receptive environment, yet this interest must be met with institutional commitment. Embedding robotic surgery within the medical student curriculum will require more than access to a simulator. It demands faculty buy in, dedicated teaching time and alignment with existing clinical learning objectives.

As robotic surgery becomes a routine feature of surgical care, there is a risk that medical students will be left behind. Early exposure to robotic technology has been shown to increase confidence, clinical readiness, and interest in a surgical career [23]. These benefits extend beyond those pursuing surgery. Understanding robotic procedures helps all medical students counsel patients, engage in interprofessional teamwork, and feel prepared for modern surgical environments [47]. However, access to robotic education remains unbalanced. Robotic systems are expensive and simulation tools are often prioritised for vocational training. Without deliberate planning, this may deepen disparities between institutions, creating unjust learning opportunities based on geography or available resources.

Comprehensive training on robotic surgery is still lacking and needs greater prioritisation. A validated, structured curriculum for surgical trainees has yet to be established. Many registrars report limited access to robotic platforms and inadequate intraoperative teaching during their training years [1, 2]. In this context, the idea of stretching limited resources to include medical students is often met with scepticism from hospitals, faculty, and even industry partners [1, 2]. However, advances in simulation, virtual reality, and telepresence platforms like Proximie offer scalable, low-risk opportunities for student learning [48]. These tools reduce reliance on theatre access and allow students to gain foundational understanding without competing with trainees for operative time. Rather than diluting training, incorporating students may actually enrich the learning environment, normalising the technology and fostering a culture of curiosity, collaboration and technological literacy from the earliest stages of clinical education.

Limitations.

This scoping review has several limitations. First, only studies published in English were included, which may have excluded relevant non-English literature. Second, as is standard in scoping review methodology, no formal assessment of study quality was performed. This limits the ability to evaluate the robustness of the included evidence. Third, most studies were small, single-institution pilots and varied considerably in structure, content, and outcome measures. This heterogeneity limited direct comparisons and made it difficult to identify best practices. Additionally, there is a risk of publication bias, as unpublished curricula or institutional programs without peer-reviewed evaluation may have been missed. Despite these limitations, this review provides a valuable overview of the current state of robotic surgery education for medical students and highlights critical gaps and opportunities for future curriculum development.

## Conclusion

Robotic surgery is rapidly becoming a standard component of surgical care. However, medical education has yet to reflect this shift in a consistent or structured way. While early pilot programs demonstrate that robotic curricula for medical students are feasible, acceptable and educationally valuable, these efforts remain limited and disconnected. There is strong interest among students and increasing support in the literature for structured, student-focused curricula. To move forward, institutions must invest not only in simulation access but also in faculty development and curriculum design. Early exposure to robotic systems offers an opportunity to build foundational knowledge, foster non-technical skills, and inspire the next generation of clinicians, regardless of their chosen specialty. By engaging students at this stage, we can better prepare the next generation of doctors for a future where robotic surgery is routine clinical practice. This review underscores the need for a coordinated, evidence-informed approach to integrating robotic surgery into medical student education.

## Data Availability

No new data were generated or analysed during this study. All relevant data are included in the article.

## Appendix 1: Medline Search Strategy

**Table:**

#	Query	Results from 15 Apr 2024
1	medical student*.mp. or Students, Medical/ or Education, Medical, Undergraduate/ or undergraduate*.mp	523,948
2	Robotic Surgical Procedures/ed [Education]	662
3	Robotics/ed [Education]	468
4	exp Surgical Procedures, Operative/ed or exp Specialties, Surgical/ed	44,408
5	3 and 4	369
6	2 or 5	1,000
7	1 and 6	80
8	robot* surg*.mp	52,093
9	1 and 8	758
10	7 or 9	764
11	Robotic Surgical Procedures/ or ((exp Surgical Procedures, Operative/ or exp Specialties, Surgical/) and Robotics/)	77,369
12	1 and 11	1,044
13	10 or 12	1,274
14	robot*.mp	223,029
15	surg*.mp	9,612,002
16	1 and 14 and 15	1,495
17	13 or 16	1,523

## Appendix 2: Data Extraction Template

**Table:**

General information
Title
Title of paper / abstract / report that data are extracted from
Lead author
Date of publication
Country in which the study conducted
United States
UK
Canada
Australia
Other
Notes
Characteristics of included studies
Methods
Aim of study
Study design
Randomised controlled trial
Non-randomised experimental study
Cohort study
Cross sectional study
Case control study
Systematic review
Qualitative research
Prevalence study
Case series
Case report
Diagnostic test accuracy study
Clinical prediction rule
Economic evaluation
Text and opinion
Other
Participants
Population description
Total number of participants
Outcome
Primary outcome
Relevant secondary outcomes
Themes
Main topic(s)
Kern framework
Problem Identification and General Needs Assessment
Targeted Needs Assessment
Goals and Objectives
Educational Strategies
Implementation
Evaluation and Feedback
Key points from paper to inform curriculum development

## Appendix 3: PRISMA-ScR checklist

**Table:**

Section/Topic	Reported Location in Manuscript
Title	Title page, line 1—Identifies the report as a scoping review
Abstract	Structured abstract includes background, objective, methods, results, and conclusion (lines 2–16)
Introduction	Introduction section, lines 17–44—Justifies the need based on technological shifts and educational gaps
Objectives	Introduction, lines 45–48—Clear aim stated: to map curricula and evaluate against Kern’s framework
Eligibility criteria	Methods, 'Eligibility Criteria' subheading—Lists inclusion/exclusion criteria and rationale
Information sources	Methods, 'Information Sources and Search Strategy'—MEDLINE, EMBASE, Cochrane; manual citation searching
Search	Appendix 1—Complete Medline search strategy included
Selection of sources of evidence	Methods, 'Selection Process'—Dual reviewer screening with consensus process
Data charting process	Methods, 'Data Extraction and Analysis'—Use of jointly created charting form
Data items	Appendix 2—Lists all extracted data fields and Kern alignment
Critical appraisal	Not applicable—No formal quality assessment performed (acknowledged in Limitations)
Synthesis of results	Methods and Results—Data grouped and summarised under Kern framework themes
Results – Selection	Results opening paragraph and PRISMA flowchart (Fig. 2)—Reports screening, exclusions, and final inclusions
Results – Characteristics	Table 2—Study design, country, sample size, aims, and outcomes detailed
Results – Charting	Table 3—Key data extracted to inform curriculum development under Kern categories
Results – Summary	Final Results paragraphs—Summary of trends, gaps, and educational strategies
Discussion – Summary	Discussion opening paragraphs—Summarises gaps, feasibility, learner enthusiasm
Discussion – Limitations	Limitations section—English-only, small studies, no quality appraisal
Discussion – Conclusions	Conclusion section—Highlights need for structured curricula, future directions
Funding	Funding section—Notes government scholarship support and author roles
